# Impaired telomere pathway and fertility in Senescence-Accelerated Mice Prone 8 females with reproductive senescence

**DOI:** 10.18632/aging.204731

**Published:** 2023-05-23

**Authors:** Alba M. Polonio, Marta Medrano, Lucía Chico-Sordo, Isabel Córdova-Oriz, Mauro Cozzolino, José Montans, Sonia Herraiz, Emre Seli, Antonio Pellicer, Juan A. García-Velasco, Elisa Varela

**Affiliations:** 1 IVI Foundation, The Health Research Institute La Fe (IIS La Fe), Valencia, Spain; 2 IVIRMA Rome, Rome, Italy; 3 Centro Anatomopatológico, Madrid, Spain; 4 IVIRMA New Jersey, Basking Ridge, NJ 07920, USA; 5 Department of Obstetrics, Gynecology and Reproductive Sciences, Yale School of Medicine, New Heaven, CT 06510, USA; 6 Department of Pediatrics, Obstetrics and Gynecology, University of Valencia, Valencia, Spain; 7 IVIRMA Madrid, Madrid, Spain; 8 Department of Obstetrics and Gynecology, Rey Juan Carlos University, Madrid, Spain

**Keywords:** telomere, telomerase, aging, fertility, ovary, SAMP8

## Abstract

Ovarian aging is the main cause of infertility and telomere attrition is common to both aging and fertility disorders. Senescence-Accelerated Mouse Prone 8 (SAMP8) model has shortened lifespan and premature infertility, reflecting signs of reproductive senescence described in middle-aged women. Thus, our objective was to study SAMP8 female fertility and the telomere pathway at the point of reproductive senescence. The lifespan of SAMP8 and control mice was monitored. Telomere length (TL) was measured by *in situ* hybridization in blood and ovary. Telomerase activity (TA) was analyzed by telomere-repeat amplification protocol, and telomerase expression, by real-time quantitative PCR in ovaries from 7-month-old SAMP8 and controls. Ovarian follicles at different stages of maturation were evaluated by immunohistochemistry. Reproductive outcomes were analyzed after ovarian stimulation. Unpaired *t*-test or Mann-Whitney test were used to calculate *p*-values, depending on the variable distribution. Long-rank test was used to compare survival curves and Fisher’s exact test was used in contingency tables. Median lifespan of SAMP8 females was reduced compared to SAMP8 males (*p* = 0.0138) and control females (*p* < 0.0001). In blood, 7-month-old SAMP8 females presented lower mean TL compared to age-matched controls (*p* = 0.041). Accordingly, the accumulation of short telomeres was higher in 7-month-old SAMP8 females (*p* = 0.0202). Ovarian TA was lower in 7-month-old SAMP8 females compared to controls. Similarly, telomerase expression was lower in the ovaries of 7-month-old SAMP8 females (*p* = 0.04). Globally, mean TL in ovaries and granulosa cells (GCs) were similar. However, the percentage of long telomeres in ovaries (*p* = 0.004) and GCs (*p* = 0.004) from 7-month-old SAMP8 females was lower compared to controls. In early-antral and antral follicles, mean TL of SAMP8 GCs was lower than in age-matched controls (*p* = 0.0156 for early-antral and *p* = 0.0037 for antral follicles). Middle-aged SAMP8 showed similar numbers of follicles than controls, although recovered oocytes after ovarian stimulation were lower (*p* = 0.0068). Fertilization rate in oocytes from SAMP8 was not impaired, but SAMP8 mice produced significantly more morphologically abnormal embryos than controls (27.03% in SAMP8 vs. 1.22% in controls; *p* < 0.001). Our findings suggest telomere dysfunction in SAMP8 females, at the time of reproductive senescence.

## INTRODUCTION

Life expectancy has increased during the last decades [1], but women’s reproductive lifespan remains unchanged [2, 3]. This fact has implications both for fertility and elderly health. Firstly, fertility is currently threatened due to socioeconomic factors which motivate couples to delay or even decline parenthood [4], and, secondly, elderly health is compromised, as menopause onset is linked to higher risks of aging-associated diseases [5, 6].

Aging can be defined as the gradual, time-dependent loss of physiological integrity, due to the accumulation of cellular damage which leads to impaired regenerative capacity of tissues and increased susceptibility to disease and death [7]. Telomere attrition has been identified as one of the molecular determinants of aging [7]. Telomeres are nucleoprotein structures composed of a repetitive six-nucleotide (5′TTAGGG3′) DNA sequence, localized at the ends of eukaryotic chromosomes, to prevent chromosome ends from being recognized as DNA breaks and protecting them from DNA repair activities and degradation [8]. However, telomeres shorten during cell divisions because DNA polymerases cannot copy the very ends of chromosomes [9]. The accumulation of critically short telomeres leads to cellular senescence or apoptosis [10, 11], limiting the regenerative capacity of tissues [12]. Telomere shortening is associated with aging [7, 13], and individuals with shorter mean TL than average for their age, have a higher risk of aging-associated diseases [14, 15] and mortality [16]. Telomere shortening can be counteracted by the action of telomerase, a ribonucleoprotein enzyme composed of a reverse transcriptase protein component (*Tert*) and an RNA component (*Terc*), which serves as a template for telomere elongation [17]. In telomerase-deficient mice, the accumulation of short telomeres causes defects in stem cell functionality [18–20] leading to accelerated aging [21, 22] and shortened lifespan [23, 24]. Also, telomerase mutations in humans, result in the so-called telomere syndromes, such as, dyskeratosis congenita [25, 26], aplastic anemia [27], or pulmonary fibrosis [28], showing similar phenotypes to telomerase-deficient mice [15, 19]. In natural conditions, telomerase is detectable in adult and embryonic stem cells, cancer cells and in the germ line [29, 30]. Among ovarian cell types, TA is found in oocytes [31, 32], and granulosa [33, 34] and cumulus cells [35]. Nevertheless, ovaries age at a faster pace compared to other organs [2, 3]. Indeed, ovarian aging is one of the main causes of infertility, characterized by the reduction of both the quantity and the quality of gametes, starting at about mid-thirties and leading to menopause at an average age of 50 years [2–4, 36]. In line with the notion that telomeres are linked to infertility, short telomeres in polar bodies extruded from oocytes are associated with an increased risk of embryo aneuploidy [37]. In addition, decreased TL and low or null TA in GCs and peripheral blood mononuclear cells (PBMCs) are found in women with premature ovarian failure [38–41].

Mouse models resembling the ovarian function decay of middle-aged women are scarce. Among those, Senescence-Accelerated Mice Prone 8 (SAMP8), a spontaneous animal model [42] recapitulates signs of reproductive aging in middle-aged women [43]. The SAMP8 model was generated from the AKR/J strain by selective inbred crosses of mice, based on graded scores for lifespan and senescence, along with pathologic phenotypes [44, 45]. One of the different strains with accelerated senescence, SAMP8 model, displays immune dysfunction [46], altered circadian rhythms [47], behavioral and emotional alterations [48], and memory and learning impairment [44, 48–50], with milder defects in females [49, 51]. SAMP8 has also been proposed as a model of Alzheimer’s disease at senectitude [42, 52]. Regarding fertility, the hypothalamus-pituitary-ovary axes is altered in SAMP8 females [53, 54], which, at 7 months of age, have shortened estrous cycles, high levels of FSH, and lower fertility [43]. The concurrence of all these symptoms, which have an early onset compared with SAMP8 lifespan, is similar to middle-aged women’s reproductive aging [43].

In addition, SAMP8 has shorter lifespan compared to the control senescence-accelerated mouse resistant 1 (SAMR1) mice [52], which do not have reproductive senescence. Interestingly, several characteristics found in the SAMP8 model are similar to those found in the second and third generation of telomerase-deficient mice, which besides accelerated telomere shortening and reduced lifespan [19, 23, 52] also show spindle aberrations in their oocytes [43, 55], reduced fertility [43, 56], or even fertility loss [19, 43].

In the current study, we sought to investigate whether the SAMP8 mice, which show accelerated-reproductive senescence have alterations in their telomere pathway. This question has not yet been explored in relation to reproduction in this model. We found alterations in the telomere pathway coinciding with fertility disorders in 7-month-old (29 weeks) female SAMP8 mice, at a time point when the survival was not different compared to controls.

## RESULTS

### SAMP8 females have shorter lifespan

**Figure 1:**
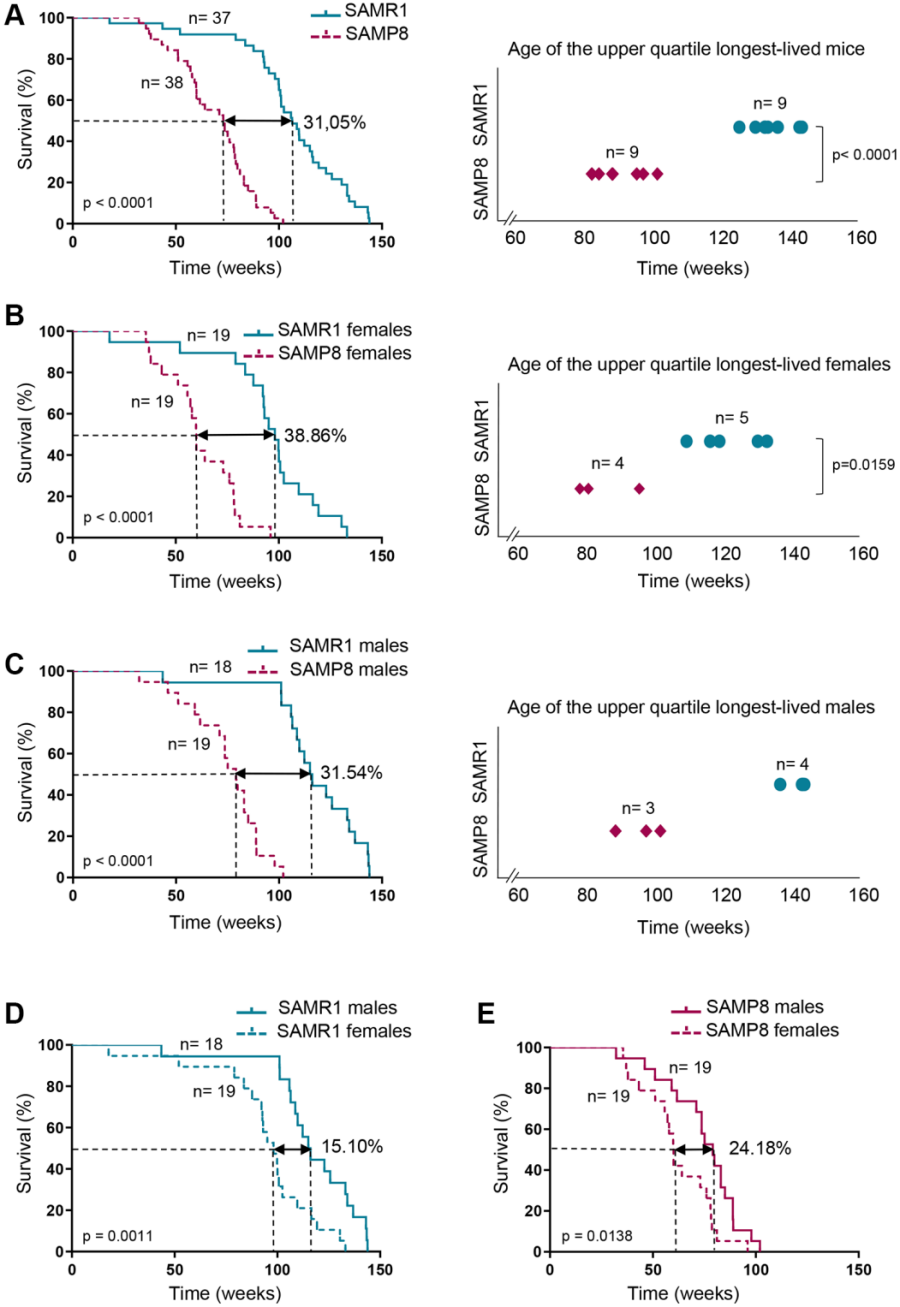
**Analysis of SAMP8 and SAMR1 lifespan.** (**A**) Kaplan-Meier plot of survival of SAMP8 (purple lines) and SAMR1 (turquoise lines) in the left panel, and graphic representation of the time of death of the Q3 longest-lived SAMP8 (purple rhombus) and SAMR1 (turquoise dots) mice in the right panel. (**B**) Kaplan-Meier plot of survival (left panel) and graphic representation of the time of death of the Q3 longest-lived females (right panel) of the mouse models described in A. (**C**) Kaplan-Meier plot of survival (left panel) and graphic representation of the time of death of the Q3 longest-lived males (right panel) of the mouse models described in A. (**D**) Kaplan-Meier plot of survival of SAMR1 mice breakdown by sex (females in dashed line and males in continued line). (**E**) Kaplan-Meier plot of survival of SAMP8 mice breakdown by sex (females in dashed line and males in continued line). n indicates the number of mice analyzed. Long-rank test was used to calculate *p*-values comparing lifespan distributions (**A**, **B** and **C**, left panels; **D** and **E**). *t*-test was used to determine *p*-value when comparing maximum lifespan of the upper quartile longest-lived mice (**A**, right panel). Mann-Whitney *U* test was used to determine *p*-value in maximum lifespan of the upper quartile longest-lived mice (**B** and **C**, right panels).

The lifespan of the SAMP8 mice is shorter than that of the SAMR1 mice [52]. In order to validate the lifespan of SAMP8 and SAMR1 in our housing conditions and to analyze the survival of SAMP8 and SAMR1 females, which has not been shown, we monitored 38 SAMP8 mice and 37 SAMR1 mice under free-intervention conditions. The age of death of mice involved in survival analysis is shown in Supplementary Table 1. Pathologies found after necropsy analysis in the SAMP8 and SAMR1 mice are shown in Supplementary Figures 1 and 2 and described in Supplementary Table 2. SAMP8 presented a 31.05 % shortened median lifespan (*p* < 0.0001) compared to SAMR1 (Table 1 and Figure 1A). Next, we considered males and females separately, to further discern differences in survival distributions of both models. SAMP8 females showed a decreased median lifespan (Table 1 and Figure 1B, left panel) compared to gender-matched controls (60 weeks in SAMP8 vs. 98.14 weeks in SAMR1), reaching a difference of 38.86% (*p* < 0.0001). The ages of death of the upper-longevity quartile were also statistically significantly decreased in SAMP8 compared to SAMR1 females (Figure 1B, right panel; *p* = 0.015). Similarly, SAMP8 males had shorter median lifespan (31.54%; *p* < 0.0001) compared to SAMR1 males (Table 1 and Figure 1C). Comparisons of female and male survival (Table 1 and Figure 1D and 1E) showed that in both SAMR1 and SAMP8 models, females presented a shortened median lifespan compared to males (24.18% in SAMP8 model vs. 15.10% in SAMR1 model). Together our results show that SAMP8 females have the shortest median and maximum survival among the different groups analyzed.

**Table 1:** Lifespan analysis in SAMP8 and SAMR1 models.

	**SAMR1**	**SAMP8**	**SAMR1 Females**	**SAMP8 Females**	**SAMR1 Males**	**SAMP8 Males**
**No of individuals (*n*)**	37	38	19	19	18	19
**Lifespan (weeks)**						
Median	106.4	73.36	98.14	60.0	115.6	79.14
(95% CI)	(100.0–116.1)	(59.86–78.71)	(87.71–109.7)	(51.14–78.00)	(106.4–134.0)	(61.71–88.86)
Mean ± SD	105.6 ± 27.00	68.44 ± 18.33	94.93 ± 26.23	62.14 ± 16.86	116.8 ± 23.56	74.75 ± 17.95
*p*-value	−	<0.0001^1^	−	<0.0001^1^	−	<0.0001^1^
*p*-value	−	−	(0.0009)^1^	(0.0319)^2^	−	−
**Maximum survival (weeks)**						
Age at death	143.9	102.0	133.1	96.0	143.9	102.0

*p*-value was calculated between SAMP8 and SAMR1 groups (globally, females and males). Brackets indicate *p*-value calculated between females and males of the same model. ^1^*p*-value was calculated using Mann-Whitney *U* test (groups with non-normal distribution of data). ^2^*p*-value was calculated using Student *t*-test (groups with normal distribution of data).

### Telomere maintenance is altered in 7-month-old SAMP8 females

**Figure 2:**
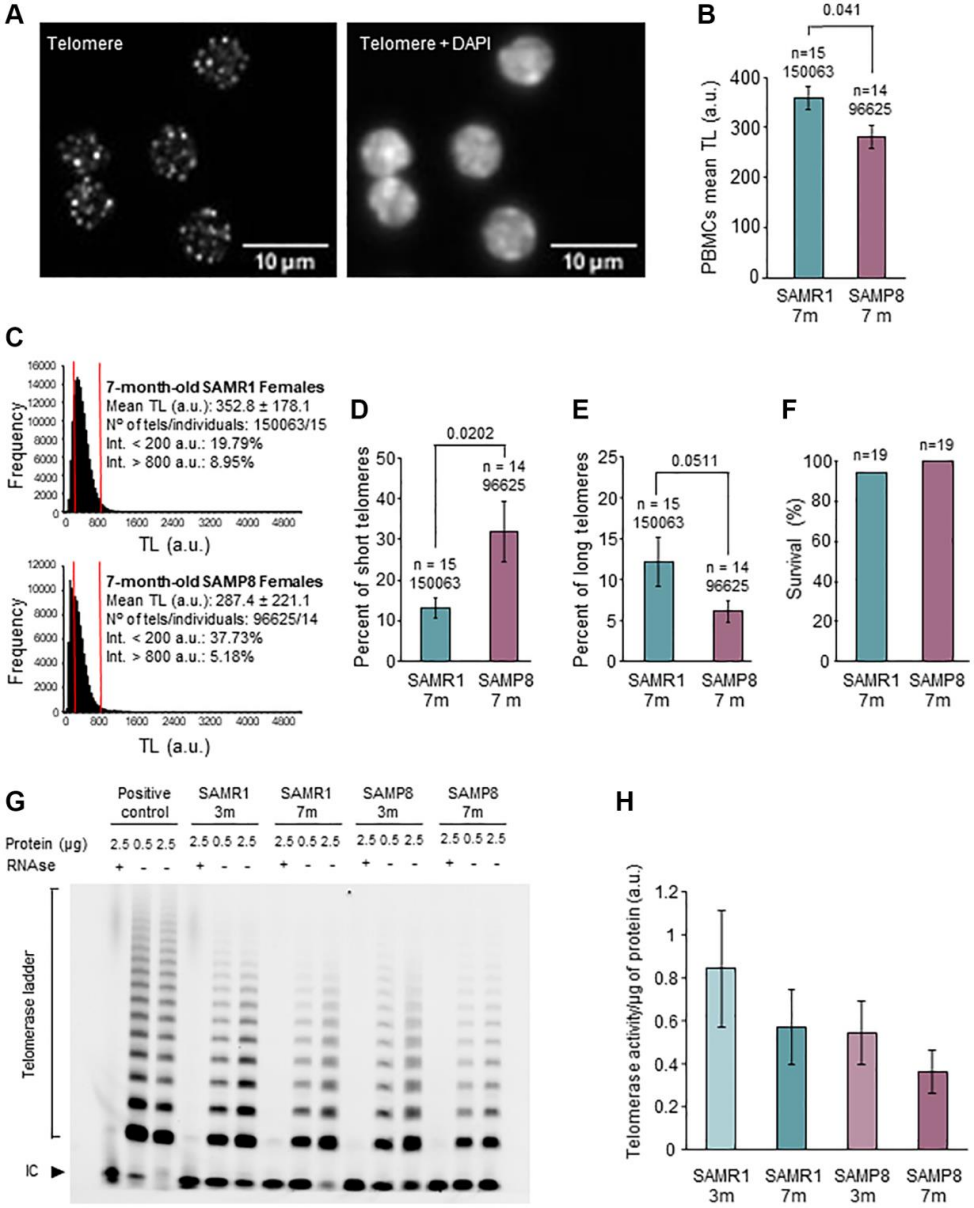
**Analysis of telomere length in PBMCs and telomerase activity in ovary.** (**A**) The micrographs show representative images of telomere HT-qFISH (white dots, left panel) and the merge (DAPI and telomeres, right panel) on PBMCs. (**B**) Mean TL of PBMCs, analyzed by HT-qFISH, in 7-month-old SAMP8 and SAMR1 females. (**C**) Telomere-length frequency histograms in 7-month-old controls (top panel) and age-matched SAMP8 females (lower panel). (**D**) Percent of short telomeres in PBMCs of 7-month-old SAMP8 and SAMR1 females. (**E**) Percent of long telomeres in PBMCs of 7-month-old SAMP8 and SAMR1 females. (**F**) The graph shows the percent of survival at 7 months of age in SAMP8 females and controls. (**G**) The micrograph shows telomerase activity assay from ovarian extracts of SAMP8 and SAMR1 females at 3 and 7 months of age. Two protein concentrations (0.5 and 2.5 μg) of the same ovarian extract from each mouse are shown. (**H**) Quantification of the telomerase activity TRAP assay shown in G. n indicates the number of mice analyzed. Underneath, the number of telomere spots analyzed is indicated. The S.E.M. is represented in error bars (**B**, **D**, **E** and **H**). Statistical significance was determined Mann-Whitney *U* test (**B**, **D** and **E**) and Fisher’s exact test (**F**). Abbreviation: IC: Internal Control. Scale bars are 10 μm.

Because SAMP8 mice had a survival curve enclosed between the curves of telomerase-deficient mice of the second and third generation [23] we next analyzed TL in PBMCs (Figure 2A). TL analysis (Figure 2B and 2C) showed that, at 7 months of age, SAMP8 females presented a statistically significant decrease in mean TL compared to age-matched controls (281.1 a.u. in SAMP8 vs. 359 a.u. in SAMR1, *p* = 0.041). Of note, no differences in mean TL were found between 3-month-old SAMP8 females and age-matched SAMR1 (Supplementary Figure 3A and 3B), or 3- or 7- month-old SAMP8 males and their age-matched controls (Supplementary Figure 3C–3F). Critically short telomeres are important because they limit cell division, leading to tissue regeneration impairment and shorter lifespan [10]. Comparing the accumulation of critically short telomeres (10^th^ percentile) in PBMCs [57], we found that 7-month-old SAMP8 females presented a statistically significantly higher percentage of critically short telomeres (Figure 2D) than age-matched SAMR1 females (32.03% in SAMP8 vs. 13.21% in SAMR1; *p* = 0.0202). In line with this result, the mean percentage of long telomeres (90^th^ percentile) was lower in 7-month-old SAMP8 females (6.128% in SAMP8 vs. 12.18% in SAMR1, *p* = 0.0511) although it did not reach statistical significance (Figure 2E). Interestingly, these differences in TL did not correspond to differences in the probability of survival (100% in SAMP8 in 94.74% in SAMR1; *p* value > 0.999) of SAMP8 and SAMR1 females, at the age of 7 months (Figure 2F). Together our results suggest that the telomere pathway is altered in females of the SAMP8 mouse model at an age of 7 months, when survival is similar for both models.

**Figure 3:**
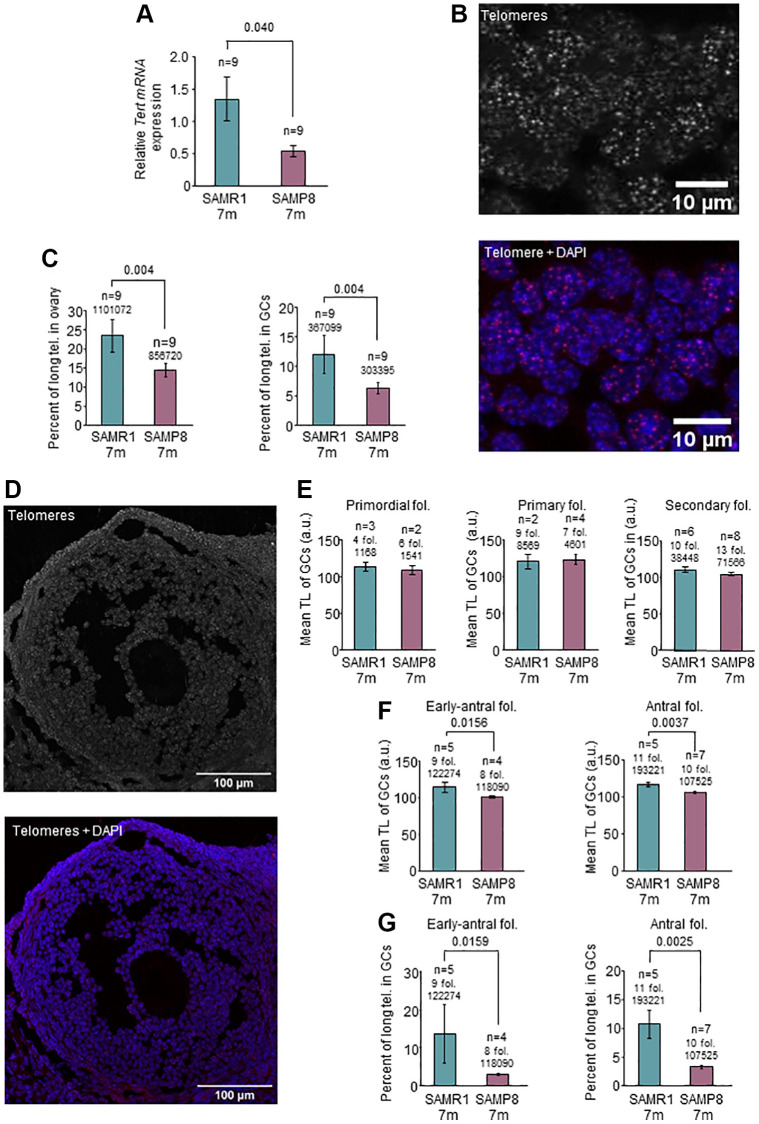
**Analysis of *Tert* expression and telomere length in the ovary.** (**A**) The graph shows mean mRNA expression of *Tert* normalized by *Gapdh* in the ovary of 7-month-old SAMP8 and SAMR1 females, measured by RT-qPCR. (**B**) The micrographs show representative images of telomere FISH on ovarian sections (white dots, left panel) and the merge (DAPI in blue and telomeres in red, right panel). (**C**) Percent of long telomeres in the ovary (left panel) and in GCs (right panel) in 7-month-old SAMP8 and SAMR1 females. (**D**) The micrographs show representative images of an antral follicle after telomere FISH (white dots, top panel) and the merge (DAPI in blue and telomeres in red, lower panel). (**E**) Mean TL, analyzed by FISH, of GCs of primordial, primary and secondary follicles in 7-month-old SAMP8 and SAMR1 females. (**F**) Mean TL, analyzed by FISH, of GCs of early-antral (left panel) and antral (right panel) follicles in 7-month-old SAMP8 and SAMR1 females. (**G**) Percent of long telomeres in GCs of early-antral (left panel) and antral follicles (right panel) in 7-month-old SAMP8 and SAMR1 females. n indicates the number of mice analyzed. Underneath the n or the number of follicles, the number of telomere spots is indicated. The S.E.M. is represented in error bars (**A**, **C**, **E**, **F** and **G**). Statistical significance was determined by unpaired *t*-test (**E** and **F**, right panels) and Mann-Whitney *U* test, for the rest of graphs. Scale bars are 10 μm (**B**) and 100 μm (**D**).

### Ovarian telomeres are altered in 7-month-old SAMP8 females

SAMP8 females have reproductive senescence at the age of 7 months [43], coinciding with systemic alterations in blood TL. Several lines of evidence point to an association between telomere alterations and fertility disorders [34, 55, 56]. Thus, we investigated telomere pathway in the ovary. We first explored TA by TRAP assay and found lower levels in SAMP8 females at the age of 7 months (Figure 2G and 2H) compared to age-matched SAMR1 and young females. Comparing both models at the age of 7 months, 73.33% of SAMP8 females showed lower TA levels in ovarian samples (*n* = 15). We then measured the levels of *Tert* expression, which correlate with TA [58]. In the ovaries of 7-month-old SAMP8 females, *Tert* expression was statistically significantly lower compared to SAMR1 females (0.543 in SAMP8 vs. 1.348 in SAMR1, *p* = 0.040) (Figure 3A). In order to determine if lower *Tert* expression would have an impact on telomere maintenance, TL in ovaries was measured (Figure 3B). Statistically significant differences were not found in mean TL either globally or in GCs (Supplementary Figure 4, Table 2). However, 7-month-old SAMP8 females presented a statistically significantly lower percentage of long telomeres (Figure 3C, Table 2) in both global ovarian tissue (6.65 in SAMP8 vs. 11.11% in SAMR1, *p* = 0.04) and GCs (6.23% in SAMP8 vs. 11.92% in SAMR1, *p* = 0.04). Although higher percentages of short telomeres were systematically found in SAMP8 samples, they did not reach statistical significance (Table 2). Given that GCs intensely proliferate during folliculogenesis, we analyzed separately GCs from each follicular stage (Figure 3D). Mean TL of GCs in primordial, primary and secondary follicles, was not different in SAMP8 and SAMR1 ovaries (Figure 3E, Table 2). Interestingly, mean TL was statistically significantly decreased in 7-month-old SAMP8 females in GCs from early-antral (100.8 a.u. in SAMP8 vs. 114.0 a.u. in SAMR1, *p* = 0.0159) and antral follicles (105.9 a.u. in SAMP8 vs. 116.7 a.u. in SAMR1, *p* = 0.0037) compared to age-matched controls (Figure 3F, Table 2). In SAMP8 females (Figure 3G, Table 2), lower accumulation of long telomeres was found in early-antral (3.03% in SAMP8 vs. 13.66% in SAMR1, *p* = 0.0159) and antral follicles (3.36% in SAMP8 vs. 10.73% in SAMR1, *p* = 0.0025). An increased percentage of critically short telomeres (Table 2) was also found in antral follicles of SAMP8 females (28.78% in SAMP8 vs. 11.31% in SAMR1, *p* = 0.0225). Our results suggest that lower *Tert* expression levels and TA may impact TL of GCs in developing follicles.

**Table 2:** Telomere analysis in ovaries from 7-months old SAMP8 and SAMR1 females.

	**TL**		**Percent of short telomeres**		**Percent of long telomeres**	
	**Mean ± SD**		**Mean ± SD**		**Mean ± SD**	
	**SAMR1**	**SAMP8**	**SAMR1**	**SAMP8**	**SAMR1**	**SAMP8**
**Global ovarian tissue**	111.6 ± 8.097	106.4 ± 3.493	12.46 ± 6.584	16.06 ± 5.957	11.11 ± 7.417	6.649 ± 1.885
Number of individuals	9	9	9	9	9	9
*p*-value	0.0625^1^		0.2419^2^		0.0400^1^	
**GCs**	110.4 ± 10.40	105.0 ± 4.878	13.19 ± 10.50	15.30 ± 7.068	11.92 ± 9.690	6.226 ± 2.842
Number of individuals	9	9	9	9	9	9
*p*-value	0.1785^2^		0.3401^1^		0.0400^1^	
**GCs in primordial follicles**	113.5 ± 9.976	108.9 ± 8.627	11.76 ± 10.94	25.72 ± 15.12	11.0 ± 9.039	10.01 ± 6.474
Number of follicles	4	6	4	6	4	6
*p*-value	0.800^1^		0.400^1^		>0.9999^1^	
**GCs in primary follicles**	120.3 ± 13.22	123.1 ± 14.36	15.00 ± 17.82	28.82 ± 13.22	13.08 ± 12.21	4.378 ± 2.504
Number of follicles	9	7	9	7	9	7
*p*-value	>0.999^1^		0.2571^1^		0.1194^2^	
**GCs in secondary follicles**	110.4 ± 9.248	104.3 ± 6.679	12.21± 7.681	16.70 ± 9.482	11.09 ± 8.175	6.151 ± 3.993
Number of follicles	10	13	10	13	10	13
*p*-value	0.1743^2^		0.3627^2^		0.3450^1^	
**GCs in early-antral follicles**	114.0 ± 15.38	100.8 ± 2.421	25.66 ± 26.92	29.82 ± 5.625	13.66 ± 17.07	3.032 ± 0.7869
Number of follicles	9	8	9	8	9	8
*p*-value	0.0159^1^		0.7731^2^		0.0159^1^	
**GCs in antral follicles**	116.7 ± 6.858	105.9 ± 2.970	11.31 ± 6.418	28.78 ± 13.30	10.73 ± 5.438	3.362 ± 0.8148
Number of follicles	11	10	11	10	11	10
*p*-value	0.0037^2^		0.0225^2^		0.0025^1^	

^1^*p*-value was calculated using Mann-Whitney *U* test (groups with non-normal distribution of data). ^2^*p*-value was calculated using Student *t*-test (groups with normal distribution of data).

### Ovarian function is altered in 7-month-old SAMP8 females

**Figure 4:**
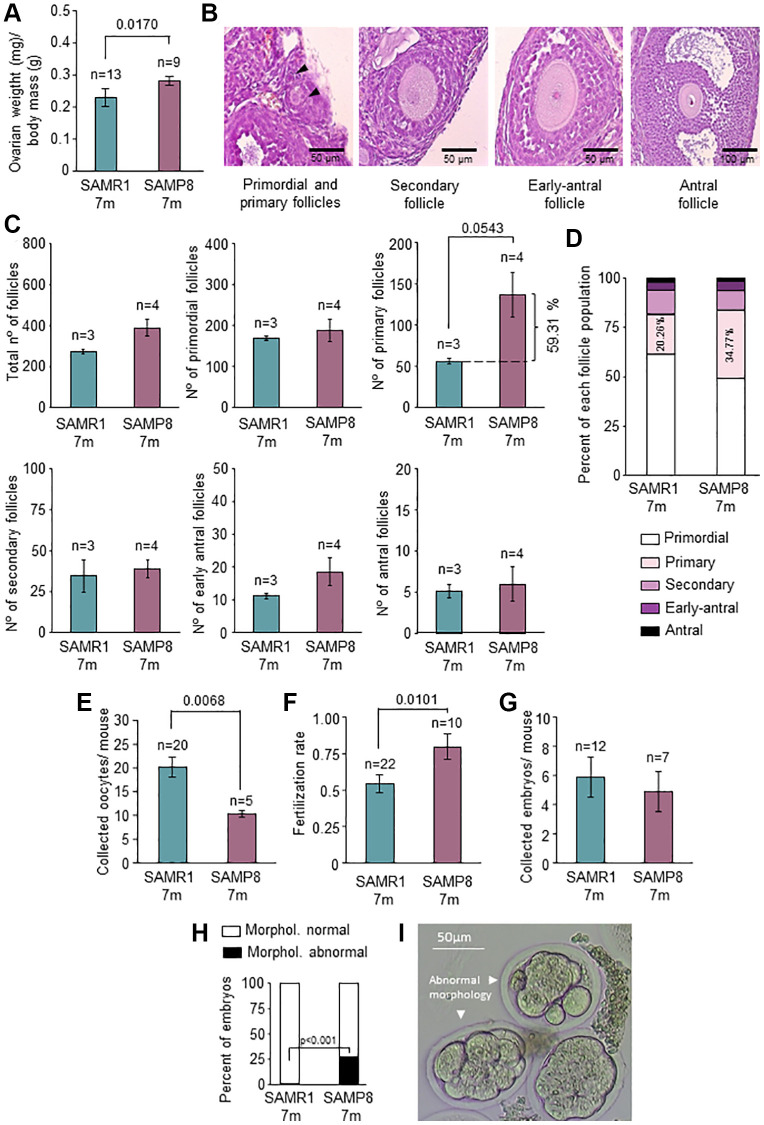
**Characterization of ovarian function and fertility.** (**A**) The graph shows the ratio of ovarian weight normalized to total body mass in 7-month-old SAMP8 and SAMR1 females. (**B**) Representative images of primordial, primary, secondary, early-antral, and antral follicles in H&E-stained ovarian sections. (**C**) Follicle count in H&E-stained ovarian sections in the mice described in A. The total number of follicles (left top panel), primordial (middle top panel), primary (right top panel), secondary (left lower panel), early-antral (middle lower panel) and antral follicles (right lower panel) are represented. (**D**) The graph shows follicle types in percentages in mice described in A. The percentages of primary follicles are indicated inside the corresponding box. (**E**) Mean number of collected oocytes after OS in mice described in A. (**F**) Fertilization rate in mice described in A. (**G**) Mean number of collected embryos after ovarian stimulation in mice described in A. (**H**) Percent of morphologically normal and abnormal embryos in mice described in A. (**I**) Representative images of preimplantation embryos (morphologically abnormal embryos are indicated with arrows). n indicates the number of mice analyzed. The S.E.M. is represented in error bars (**A**, **C**, **E**, **F** and **G**). Statistical significance was determined by Mann-Whitney *U* test (**A**, **C**, **E** and **F**) and unpaired *t*-test (**G**). Fisher’s exact test was used to determine *p*-values (**H**). Scale bars are 50 μm (**B**, left and middle panel) and 100 μm (**B**, right panel).

In telomerase-deficient mice organ function is impaired [19]. Since SAMP8 had lower TA and *Tert* levels in the ovary and lower mean TL in early-antral follicles, we tested ovarian function. Ovarian weight normalized by body mass of SAMP8 females was higher than controls (Figure 4A; *p* = 0.017), accompanied by the presence of numerous corpus luteum, found in histopathological analysis (Supplementary Figure 5). Despite the higher ovarian weight, there were not differences in absolute numbers of either total follicles or in the different follicular stages (Figure 4B and 4C). Mean number of primary follicles was higher in SAMP8 females (59.31%), although it did not reach statistical significance (Figure 4C). This was also observed when follicular stages were represented as percentages (34.77% in SAMP8 vs. 20.26% in SAMR1; *p* = 0.0567) (Figure 4D). Premature ovarian aging is reflected in the gamete production and fertility [3]. Therefore, we analyzed the number of oocytes collected (Figure 4E) after ovarian stimulation (OS), finding that 7-month-old SAMP8 females produced significantly lower number of oocytes (*p* = 0.0068). Fertilization rate was not impaired in SAMP8 females (Figure 4F) and the number of collected embryos were not different between the groups (Figure 4G). However, the percentage of morphologically abnormal embryos was significantly higher in SAMP8 females (27.03% in SAMP8 vs. 1.22% in SAMR1; *p* < 0.001; Figure 4H and 4I). Our results suggest that oogenesis and embryo development is impaired in 7-month-old SAMP8 mice compared to age-matched controls, coinciding with alterations in the telomere pathway.

## DISCUSSION

In this work, we have found, a robust phenotype, in terms of reduced lifespan, in SAMP8 females. Interestingly, at the age of 7 months, when survival was similar in both groups, TL in PBMCs was shorter and TA was decreased in SAMP8 females compared to age-matched SAMR1 female mice. In addition, in the middle-aged (7-month-old) SAMP8 females, TA, telomerase expression and the accumulation of longer telomeres in the ovaries were reduced. These changes coincided with impaired reproductive function in SAMP8 mice, with decreased number of collected oocytes after OS and higher percentage of morphologically abnormal embryos.

Regarding lifespan, the median and maximum values obtained for SAMP8 were lower than in SAMR1, as shown earlier [52]. Female mice of both models had a lower median survival compared to males, and SAMP8 females presented the shortest median and maximum lifespan of the mice studied. Our postmortem-examination findings coincide with former reports for both models [59, 60]. Interestingly, the survival curve of SAMP8 mice resembled that of telomerase-deficient mice, lying between the ones of second- and third-generations without telomerase [23], in whose absence, telomere shortening is increased on each subsequent generation [24].

Concerning telomeres, which are a primary cause of aging [7], TL was shorter in PBMCs of middle-aged SAMP8 females. Moreover, SAMP8 females accumulated more critically short telomeres, which are shown to correlate with lifespan [10]. Indeed, critically short telomeres may cause cellular senescence [7], and the accumulation of senescence cells in tissues leads to aging [61, 62]. Ultimately, TA is responsible for TL maintenance [63]. In fact, in humans, lower TA levels lead to telomere shortening and the development of severe diseases such as liver cirrhosis [64], pulmonary fibrosis [28], aplastic anemia [27] and dyskeratosis congenita [65]. These disorders concur with shorter lifespan and limited regenerative capacity of tissues [14, 15].

An association between telomeres and fertility has been evidenced in mice [19, 23, 34, 55, 56] and in women with fertility disorders, in whom TL and TA alterations have been described [33, 35, 38–41, 66]. Recently, in a case of dyskeratosis congenita, with altered TA, diminished fertility has been reported [66], with decreased oocyte production and fertilization rate, along with increased rate of aneuploidy and shorter TL in embryos [66]. Here, in SAMP8 females, with accelerated reproductive senescence [43] we observed lower TA and telomerase expression in ovaries, which are determinants of fertility outcomes [33]. Indeed, telomerase-deficient mice produce a lower number of oocytes, which have spindle abnormalities and chromosome misalignments [56]. Most embryos from telomerase-deficient mice do not reach the blastocyst stage, leading to reduced litter size [19]. The SAMP8 model also shows reduced littler size [43] and oocytes with spindle aberrations and chromosome misalignments [43].

Correct oocyte maturation needs an adequate ovarian niche. In 7-month-old SAMP8 females, ovarian weight was higher than in wild types, although the numbers of follicles were similar. The accumulation of corpus luteum in this model could be a plausible explanation, and also suggests a potential impairment of the pathways involved in corpus luteum regression [67, 68]. Unprecedently, we found a trend to the accumulation of primary follicles in SAMP8 females. This points to either more primordial follicles being recruited for follicular development or primary follicles having limitations to advance toward secondary follicles. Later in folliculogenesis, the number of antral follicles was similar in both models, however, the number of collected oocytes after OS was lower in the SAMP8 model, as previously shown [43]. Despite the straightforward fertilization competence of the 7-month-old SAMP8 oocytes, there was a trend to a lower production of embryos in SAMP8 females. Indeed, a remarkably high number of alterations in the morphology of preimplantation embryos in SAMP8 females was scored. This suggests that follicular development in the SAMP8 mice might yield mature oocytes but the molecular mechanisms that prevent aneuploidies may not function accurately. These results resemble what occurs in middle-aged women in whom not only the quantity of gametes is diminished [3], but also a higher rate of aneuploidies is found [69], particularly when TL is low in polar bodies extruded from oocytes [37].

In the context of the ovary, unexpectedly TL was similar in SAMP8 and SAMR1 mice, despite the lower TA and *Tert* expression in middle-aged SAMP8 females. However, globally, both in the ovary and GCs, the accumulation of long telomeres was lower in the SAMP8, which could be explained by the preferential action of telomerase on short telomeres [70]. In wild types, telomeres of GCs may be protected from excessive shortening [17] because of the presence of TA [31–34, 71]. In SAMP8 females, even reduced telomerase levels could still maintain telomeres at early follicular stages (primordial and primary follicles, which we found to have similar TL as controls) because cell division is limited. To reach later stages of folliculogenesis, GCs have to undergo active and repetitive cell divisions [72], and reduced telomerase may not be able to sustain wild-type levels of TL. This could explain the lower mean TL and percentage of long telomeres found in early-antral and antral follicles as well as the increment in the percentage of critically short telomeres found in antral follicles of SAMP8 mice. Thus, lower levels of telomerase in the ovary seem to impact telomere maintenance of GCs at the end of follicle development.

Overall, our results suggest that the telomere pathway is altered in middle-aged SAMP8 females not only at the systemic level (shorter telomeres in PBMCs) but also in the ovarian compartment (lower telomerase expression and activity as well as altered telomeres in GCs). All of it concurs with the onset of reproductive senesce symptoms in the SAMP8, at a time in which survival is not altered. Dysfunctions in the telomere pathway are also observed in women with fertility disorders [38, 40, 66]. In addition, our results show alterations in embryo development, which have also been associated with short telomeres in humans [37].

Understanding the molecular pathways underlying aging and fertility, provides a basis for further studies focused on several topics. First, the analysis of embryo alterations, which can be better assessed in mice than in humans. Second, how reproductive lifespan improvement may ameliorate elderly health. And third, the mechanisms underlying follicle recruitment and development, which are not completely known. Thus, SAMP8 females represent a bona fide model for the analysis of fertility, not only because it shows similar phenotype to middle-aged women as stated earlier [43], but also because the alterations in the telomere pathway are found in women with fertility disorders [37, 38, 40, 41] and this pathway links reproduction with longevity.

## METHODS

### Animal handling

All animal procedures were performed according to protocols approved by the Ethics Committee of the Rey Juan Carlos University (code 2509201913119) on 18th of November of 2019. The senescence-accelerated mice, selected from inbred crosses of the AKR/J mouse strain [44], were a kind gift of Dr. Helena Mira Aparicio (IBV, CSIC, Valencia, Spain). Mice were raised under specific-pathogen-free conditions and standard 12-h light-dark cycles in Rey Juan Carlos University Animal Production and Experimentation Service and they were provided with food and water *ad libitum*.

### Study design

Middle-aged SAMP8 females (7-months old; 29 weeks), with accelerated-reproductive senescence, and age-matched SAMR1 females, which do not have reproductive senescence (controls), were used for experiments.

### Survival analysis

SAMR1 mice (*n* = 37; 19 females and 18 males) and SAMP8 mice (*n* = 38; 19 females and 19 males) were used for lifespan analysis. All mice were maintained under intervention-free conditions. To determine the time of death, mice were inspected three times per week. Moribund mice were euthanized if they were severely ill or if the veterinarian from the Animal Production and Experimentation Service concluded that they would not survive more than 2 days. The age at which euthanasia was performed was considered as the best estimation of the time of natural death.

### Sample collection

Mice were sacrificed by inhalation of carbon dioxide (CO_2_) or by cervical dislocation (in the case of oocytes and preimplantation embryos). Ovaries were collected and fat surrounding the ovary was removed, followed by ovarian weight measurement. One ovary was frozen in liquid nitrogen and stored at −80ºC. The other ovary was fixed in 4% formaldehyde for 24 hours and treated as explained below depending on the experiments performed. Blood samples were collected by cardiac puncture in K2-EDTA tubes (BD Vacutainer) and PBMCs were isolated by using Ficoll gradient (Histopaque, Sigma), fixed with methanol: acetic acid (3:1) and stored at 4ºC. In the case of ovaries (Figures 3 and 4), samples were divided for different techniques (FISH, RT-PCR, H&E and TRAP), thus, experiments were done with lower “n” compared to results in Figure 2.

### Follicle counts

Fixed ovaries were embedded in paraffin and cut into 4 μm sections. Follicle count was performed on every fifth section stained in Hematoxylin–Eosin (H&E). Follicles were classified as previously described [73] as: (a) primordial: the oocyte was surrounded by a layer of flattened GCs; (b) primary: the oocyte was surrounded by a complete layer of cuboidal GCs; (c) secondary: the oocyte was surrounded by two or more layers of cuboidal GCs; (d) early-antral: the oocyte was surrounded by four or more layers of GCs, forming the follicular atrium; and (e) antral: follicles containing a clearly defined single antral space. To avoid double counting, follicles were only counted when the oocyte nucleus was present in the section. All H&E sections were examined by at least 2 observers.

### Real-time quantitative PCR (RT-qPCR)

Ovaries were homogenized using RNAse-free pestle and mortar. Ovarian total RNA isolation was performed using RNAeasy Micro Kit (QIAGEN) following manufacturer’s instructions. 1 μg of RNA was retrotranscribed to complementary DNA (cDNA) using iScriptcDNA Synthesis Kit (BioRad) according to manufacturer’s recommendations. RT-qPCR was performed using SsoAdvanced Universal SYBR Green Supermix (BioRad) according to manufacturer’s protocol in 7500 Fast Real-Time PCR System (Applied BioSystems) by the personnel of the Rey Juan Carlos University Genomics-Flow Cytometry Unit. The primers for the PCR amplification of *Tert* and *Gapdh* (Glyceraldehyde-3-phosphate dehydrogenase) genes are described below. *Gapdh* gene expression was used to calculate the relative expression of *Tert* gene, based on the cycle threshold (Ct).

*Gapdh*-F 5′-GCACAGTCAAGGCCGAGAAT-3′

*Gapdh*-R 5′-GCCTTCTCCATGGTGGTGAA-3′

*Tert*-F 5′-GGATTGCCACTGGCTCCG-3′

*Tert*-R 5′-TCAATTGGTAAGCTGTAAGTCTGTG-3′.

### Telomerase-repeat amplification protocol (TRAP) assay

Ovarian samples were homogenized using RNAse-free pestle and mortar and lysed as in [74]. The CY5-labelled telomerase-substrate primer (TS-primer: 5′-Cy5-AATCCGTCGAGCAGAGTT-3′, Sigma-Aldrich [75]); was elongated, and elongation products were amplified together with an internal control, as in [75]. Two protein concentrations were used for each sample (0.5 and 2.5 μg). A negative control was included by preincubating each sample extract with RNase (Roche Diagnosis) for 10 min at 30°C as in [76]. Jurkat cells were used as a positive control. Electrophoresis was run in an acrylamide: bisacrylamide 19:1 gel (Bio-Rad) using Protean II (Bio-Rad) electrophoresis chambers. Gels with Cy5 signals were imaged wet in ChemiDoc (Bio-Rad). Image Lab software (version 5.0) was used for quantification of the TRAP image shown in Figure 2.

### *In situ* hybridization fluorescence

TL was assessed in PBMCs and ovaries by fluorescence *in situ* hybridization (FISH). For PBMCs, High-Throughput Quantitative FISH (HT-qFISH) was performed using 96-well plates with clear bottom (Greiner, Bio-One). Fixed cells were attached to plates using poly-L-lysine (Sigma-Aldrich) for 30 min at 37°C and FISH was performed as previously described [57]. Fixed ovaries (see sample collection section) were frozen with OCT and 10 μm sections were cut. FISH on tissue sections was performed as in [76].

Briefly, samples were fixed with 4% formaldehyde for 2 min at room temperature (RT) and permeabilized with Pepsin (Sigma-Aldrich) for 10 min at 37°C. Subsequently, samples were dehydrated with increasing concentrations of EtOH (70%, 90% and 100% for 5 min at RT, respectively). Tel-Cy3 PNA probe (Cy3-(CCCTAA)_3_) (Panagene) was added in hybridization solution (containing 70% of deionized formamide) at a final concentration of 0.5 μg/mL. DNA denaturation was performed at 85°C followed by 2 h incubation at RT. Hybridization solution without the probe was added as a negative control. Intensive washes were performed in order to remove non-specifically bound probe. Nuclei were stained with DAPI (Invitrogen) and Vectashield (Vector Laboratories) was added as an antifading agent.

### Image acquisition and analysis

Images from HT-qFISH were acquired on an Opera High Content Screening System (PerkinElmer) as in [57], using 40×, 0.9 NA water-immersion objective at the Microscopy Unit of Spanish National Cancer Research Center (CNIO). To ensure that a minimum of 300 cells per case were scored, 40 images were acquired in each well. Images were analyzed with Acapella Software [57]. Images from ovarian sections were acquired on a Confocal TCS SP5 Leica Microscope equipped with a resonant scanner using a 63×, 1.4 NA oil-immersion objective at the Microscopy and Image Analysis Service (SMAI) of the National Hospital for Paraplegics of Toledo, Spain. DAPI and Cy3 signals were acquired on separate channels. Maximum-projection images from Z-stacks were used for quantitative analysis [77]. Quantitative data analysis was performed using Fiji (ImageJ 1.53f51) software with the assistance of the SMAI. For the detection of signals, the background noise of the maximum projections was subtracted. Maximum-projection signals were thresholded, and CY3 signals corresponding to telomeres, were detected with the “Analyze particles” command of ImageJ. DAPI signals were also detected to define the nuclear area, so that only telomere signals from inside the nuclear mask were considered [78]. The detections were saved in the ROI Manager to be transferred to the unprocessed images for intensity quantification. Mean CY3 intensity per telomeric spot was used for quantification and expressed as arbitrary units (a.u.) [78]. Telomeres from global ovarian tissue and GCs were analyzed. Follicle types were classified as described above (see follicle count section). Not all follicular types were present in each ovarian section, thus, the number of samples varied in experiments related to follicular types (Figure 3E–3G and Table 2).

### Reproductive outcomes

Middle-aged SAMP8 and SAMR1 females (7-months old) were superovulated by intraperitoneal injection of 10 I.U. of pregnant mare serum gonadotropin (PMSG, Folligon, MSD Animal Health) and 10 I.U. of human chorionic gonadotrophin (hCG, LeonVet) and mated with a fertile-young male. After 1.5 days, females were sacrificed, and oocytes were retrieved from the oviducts and the number of total collected oocytes was measured. Successful fertilization was confirmed by the presence of ≥ 2-cell embryos in maternal reproductive tract. Fertilization rate was calculated as the ratio of embryos divided by the number of oocytes and embryos collected. Preimplantation embryos were collected from the uterus at 3.5 days after mating. Data collection in terms of reproductive outcomes is variable because SAMP8 females do not always respond to OS (Figure 4E–4G).

### Statistics

All statistical analysis were performed using GraphPad Prism software (version 8). Data were presented as mean and standard error and the Shapiro-Wilk test was used to determine whether the data followed a normal distribution. Student’s *t*-test was applied to compare groups for variables that followed a normal distribution and the two-tailed Mann-Whitney *U* test was applied as a nonparametric method to analyze variables that did not follow a normal distribution. Log-rank test was applied to detect differences between survival curves. Fisher’s exact test was used to determine statistical significance for the analysis of probabilities in contingency tables. *p* values < 0.05 were considered statistically significant. The number of samples used for each experiment is indicated in the figures.

## Supplementary Materials

Supplementary Figures

Supplementary Tables
