# Supplementary material for: Impaired telomere pathway and fertility in Senescence-Accelerated Mice Prone 8 females with reproductive senescence
Source: Aging (Albany NY). 2023 May 23;15(11):4600–24. doi: 10.18632/aging.204731 (PMC10292900; doi:10.18632/aging.204731)
Supplement: Supplementary Figures [file aging-15-204731-s001.pdf]

## SUPPLEMENTARY FIGURES

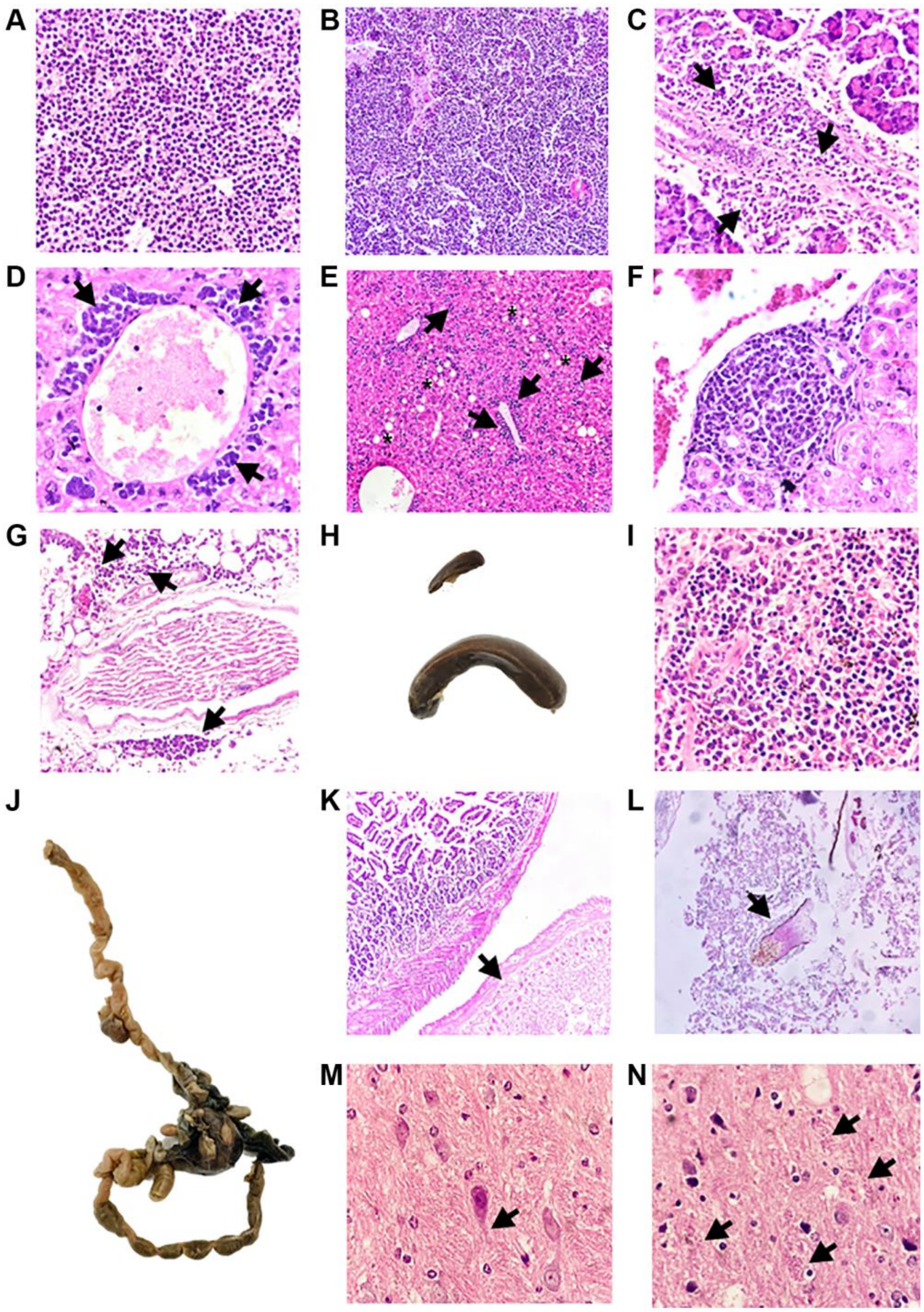


**Supplementary Figure 1. Postmortem examinations of SAMP8 mice.** Representative micrographs of necropsies in SAMP8. (**A**) Diffuse tumor infiltration of the thymus by lymphoid cells (SAMP8-18). (**B**) Lymphoid tumor with diffuse growth of cells of plasmablastic habitus in lymph node (SAMP8-07). (**C**) Lymphoid tumor lesions (indicated with arrows) in pancreatic tissue (SAMP8-07). (**D**) Lymphoid neoplasm infiltration (arrows) in hepatic perivenular areas (SAMP8-04). (**E**) Lymphoid tumor cellularity (plasmablastic, indicated with arrows) in hepatic sinusoids and perivenular areas (SAMP8-04). Asterisks indicate the presence of lipid droplets reflecting hepatic steatosis. (**F**) Lymphoid tumor lesions in kidney and (**G**) perirenal tissue (SAMP8-04). (**H**) Splenomegaly found in SAMP8 mice (SAMP8-07) (bottom) compared with a control spleen (top). (**I**) Diffuse splenic infiltration of lymphoma (SAMP8-18). (**J**) Intestinal torsion and necrosis (SAMP8-07). (**K**) Ischemic lesion (arrows) and (**L**) hairs (indicated with arrow) in feces in the intestine described in J. (**M**) Neurofibrillary tangles (indicated with arrows) in the brain (SAMPB-21). (**N**) Possible amyloid plaques (indicated with arrows) in brain (SAMP8-25).

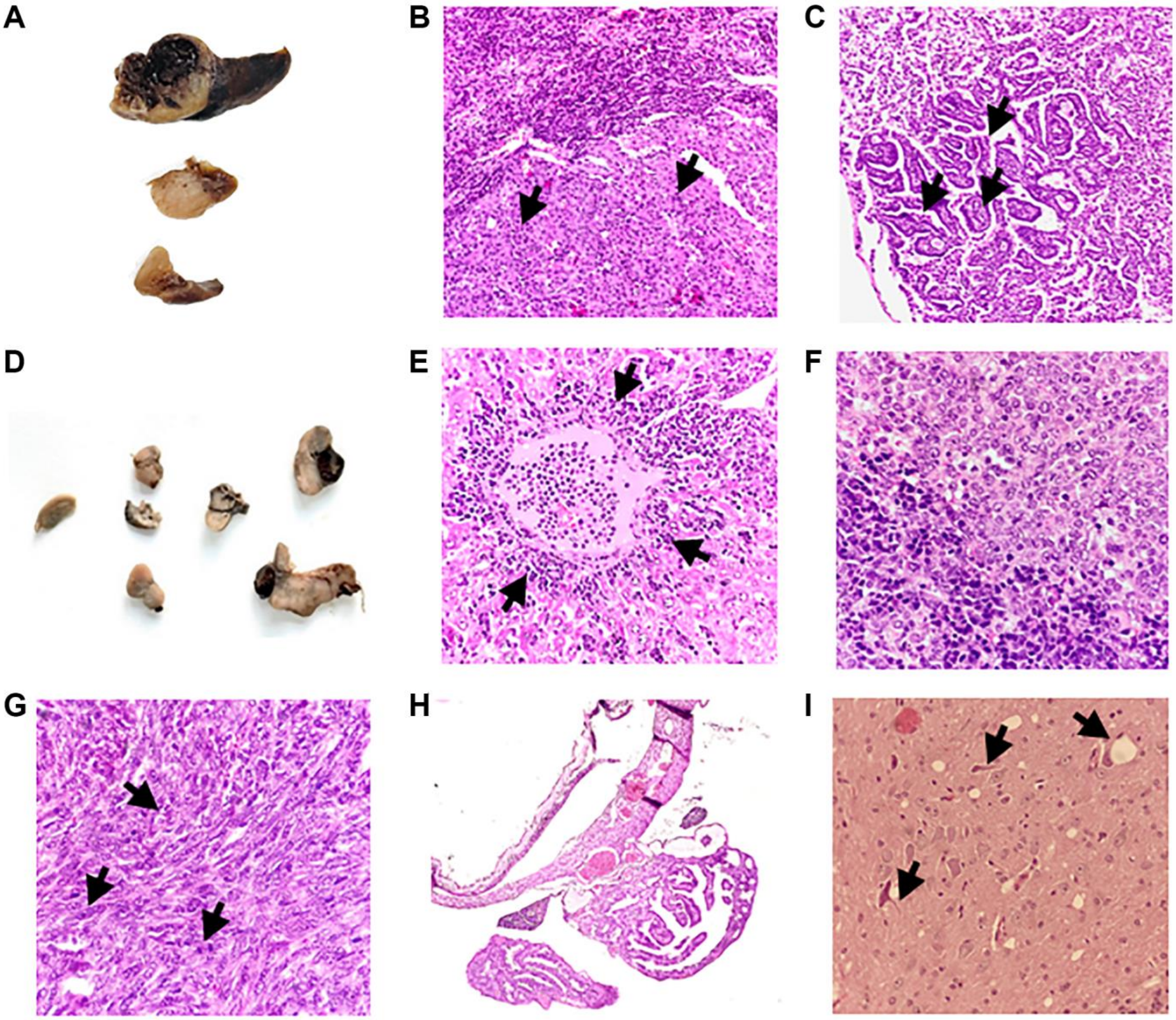


**Supplementary Figure 2. Postmortem examinations of SAMR1 mice.** Representative micrographs of necropsies in SAMR1. (**A**) Macroscopic image of a lung tumor (SAMR1-37). (**B**, **C**) Tumor foci in the lung consisting of papillary adenocarcinomas (SAMR1-37 in B and SAMR1-14 in C). Arrows indicate tumor areas. (**D**) Macroscopic image of lymph nodes thickened by tumors located adjacent to the colon, kidney and cervical region (SAMR1-07). (**E**) Metastasis of the lymphoma described in D in perivenular areas of liver, in the form of a small and large cell lymphoma (indicated by arrows). (**F**) Metastasis of the lymphoma described in D, infiltrated in spleen. Different cell morphologies are found. (**G**) Sarcoma in the abdominal wall (SAMR1 -25) consisting of a malignant spindle-cell lesion. Arrows indicate mitotic figures. (**H**) Image shows a benign cyst in peritoneum (SAMR1 -13). (**I**) Neurofibrillary tangles (indicated with arrows) in the brain (SAMR1 -25).

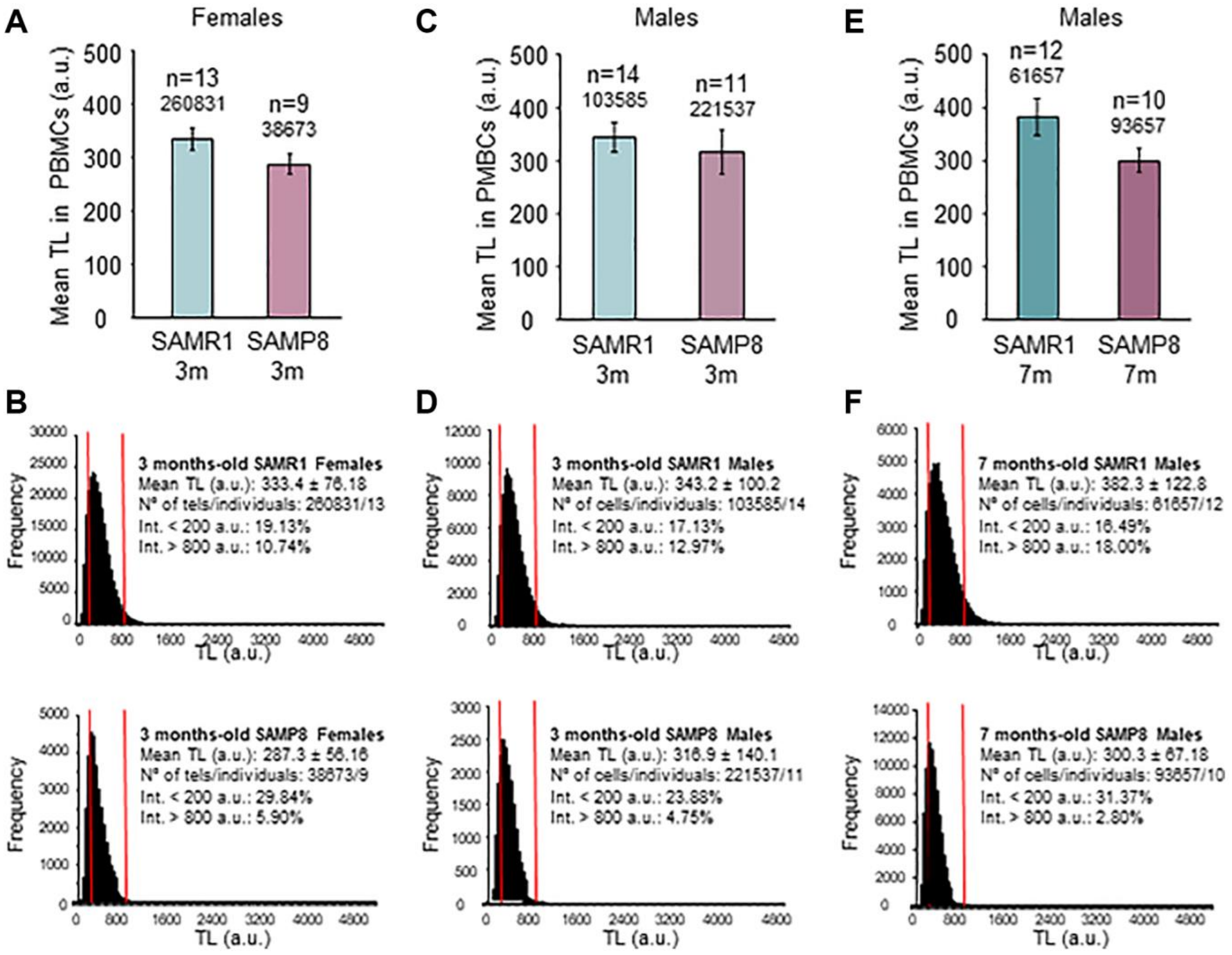


**Supplementary Figure 3. Analysis of telomere length in PBMCs.** (**A**) Mean TL of PBMCs, analyzed by HT-qFISH, in 3-month-old SAMP8 and SAMR1 females. (**B**) Telomere-length frequency histograms in 3-month-old SAMR1 (top panel) and SAMP8 females (lower panel). (**C**) Mean TL of PBMCs, analyzed by HT-qFISH, in 3-month-old SAMP8 and SAMR1 males. (**D**) Telomere-length frequency histograms in 3-month-old SAMR1 (top panel) and SAMP8 males (lower panel). (**E**) Mean TL of PBMCs, analyzed by HT-qFISH, in 7-month-old SAMP8 and SAMR1 males. (**F**) Telomere-length frequency histograms in 7-month-old SAMR1 (top panel) and SAMP8 males (lower panel). n indicates the number of mice analyzed. Underneath, the number of telomere spots analyzed is indicated. The S.E.M. is represented in error bars (**A**, **C** and **E**). Statistical significance was determined by unpaired *t*-test (**A**, **C** and **E**). *p*-values < 0.05 were considered statistically significant.

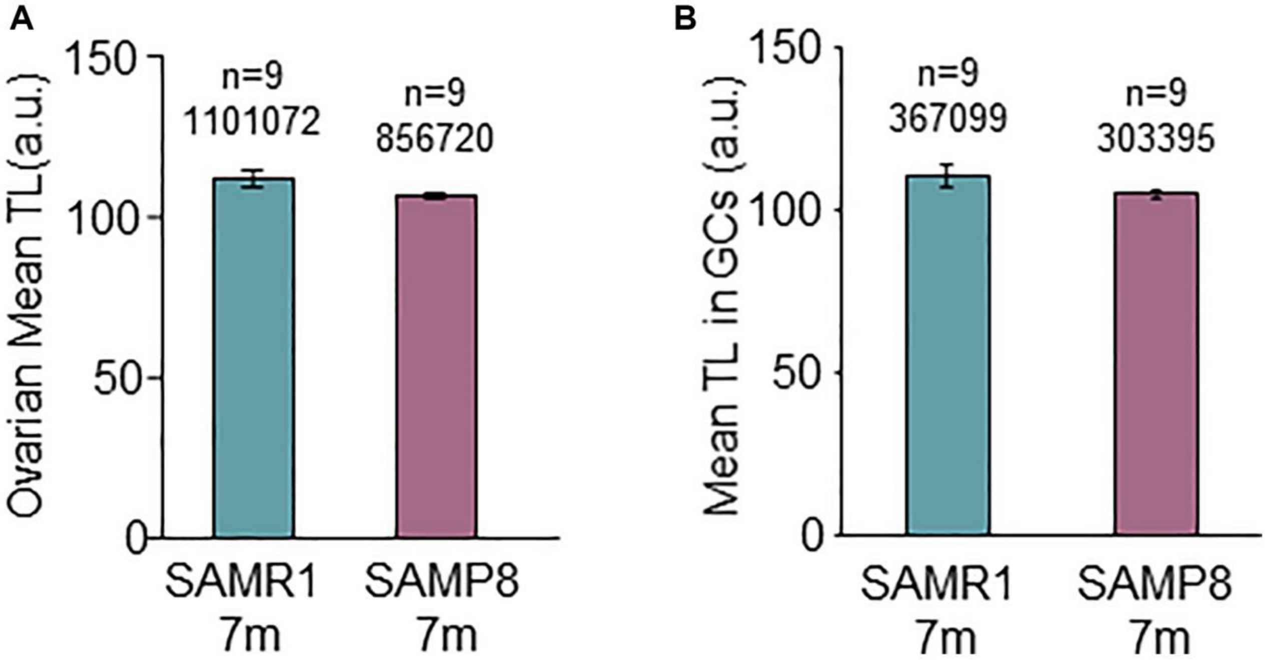


**Supplementary Figure 4. Analysis of mean telomere length in the ovary.** (**A**) Global mean TL in the ovary of 7-month-old SAMP8 and SAMR1 females, analyzed by FISH. (**B**) Mean TL in GCs of the ovary of mice described in A. n indicates the number of mice analyzed. Underneath, the number of telomere spots analyzed is indicated. The S.E.M. is represented in error bars, Statistical significance was determined by unpaired *t*-test (**B**) and Mann-Whitney *U* test (**A**). *p*-values < 0.05 were considered statistically significant.

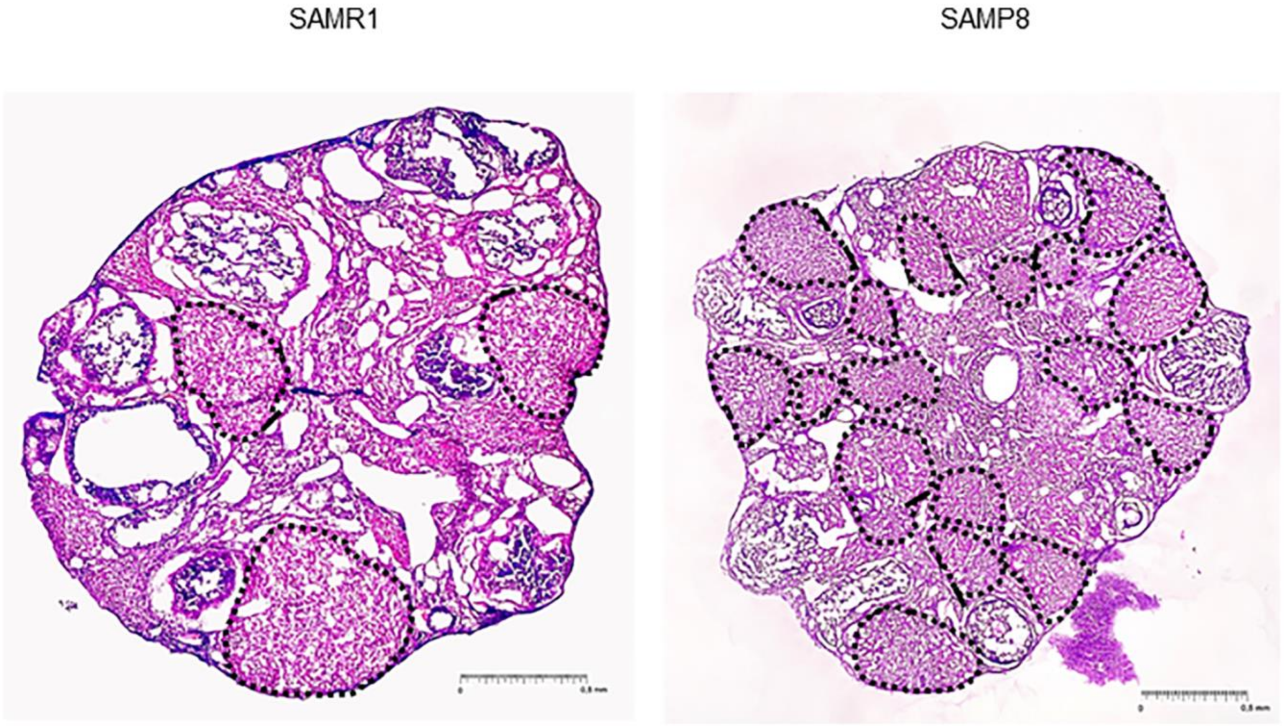


**Supplementary Figure 5. Corpus luteum in ovaries.** Representative image of ovarian sections from SAMP8 (right panel) and SAMR1 (left panel) females. Dashed lines indicate corpus luteum.
