# Supplementary material for: Impaired telomere pathway and fertility in Senescence-Accelerated Mice Prone 8 females with reproductive senescence
Source: Aging (Albany NY). 2023 May 23;15(11):4600–24. doi: 10.18632/aging.204731 (PMC10292900; doi:10.18632/aging.204731)
Supplement: Supplementary Tables [file aging-15-204731-s002.pdf]

SUPPLEMENTARY TABLES

Supplementary Table 1. Individual survival in SAMP8 and SAMR1 mice.

Mice code	Model	Sex	Age at death (weeks)
SAMP8-01	SAMP8	Female	35.57
SAMP8-02	SAMP8	Female	37
SAMP8-03	SAMP8	Female	37.86
SAMP8-04	SAMP8	Female	43.71
SAMP8-05	SAMP8	Male	51
SAMP8-06	SAMP8	Male	32.14
SAMP8-07	SAMP8	Male	61.71
SAMP8-08	SAMP8	Female	59.86
SAMP8-09	SAMP8	Female	96
SAMP8-10	SAMP8	Female	78
SAMP8-11	SAMP8	Female	73
SAMP8-12	SAMP8	Female	60
SAMP8-13	SAMP8	Female	60
SAMP8-14	SAMP8	Female	64.14
SAMP8-15	SAMP8	Female	57.86
SAMP8-16	SAMP8	Female	51.14
SAMP8-17	SAMP8	Female	76
SAMP8-18	SAMP8	Female	81.14
SAMP8-19	SAMP8	Female	57.14
SAMP8-20	SAMP8	Female	55.71
SAMP8-21	SAMP8	Female	78.71
SAMP8-22	SAMP8	Male	88.86
SAMP8-23	SAMP8	Male	71.14
SAMP8-24	SAMP8	Male	88.86
SAMP8-25	SAMP8	Male	97.86
SAMP8-26	SAMP8	Male	89
SAMP8-27	SAMP8	Male	83
SAMP8-28	SAMP8	Male	46
SAMP8-29	SAMP8	Male	79.14
SAMP8-30	SAMP8	Male	73.71
SAMP8-31	SAMP8	Male	73.71
SAMP8-32	SAMP8	Male	83
SAMP8-33	SAMP8	Male	102
SAMP8-34	SAMP8	Male	79.86
SAMP8-35	SAMP8	Male	85
SAMP8-36	SAMP8	Male	75.14
SAMP8-37	SAMP8	Male	59.14
SAMP8-38	SAMP8	Female	78.29

SAMR1-01	SAMR1	Female	17.86
SAMR1-02	SAMR1	Male	43.57
SAMR1-03	SAMR1	Female	52
SAMR1-04	SAMR1	Female	99.86
SAMR1-05	SAMR1	Female	119.29
SAMR1-06	SAMR1	Female	102.43
SAMR1-07	SAMR1	Female	100.71
SAMR1-08	SAMR1	Female	109.71
SAMR1-09	SAMR1	Female	116.57
SAMR1-10	SAMR1	Female	92.71
SAMR1-11	SAMR1	Female	92.29
SAMR1-12	SAMR1	Female	87.71
SAMR1-13	SAMR1	Female	83.71
SAMR1-14	SAMR1	Female	130.43
SAMR1-15	SAMR1	Female	100
SAMR1-16	SAMR1	Female	93
SAMR1-17	SAMR1	Female	79
SAMR1-18	SAMR1	Female	95.14
SAMR1-19	SAMR1	Female	98.14
SAMR1-20	SAMR1	Female	133.14
SAMR1-21	SAMR1	Male	112.29
SAMR1-22	SAMR1	Male	125.71
SAMR1-23	SAMR1	Male	109.86
SAMR1-24	SAMR1	Male	143.14
SAMR1-25	SAMR1	Male	108.71
SAMR1-26	SAMR1	Male	133.14
SAMR1-27	SAMR1	Male	143.86
SAMR1-28	SAMR1	Male	134
SAMR1-29	SAMR1	Male	116.14
SAMR1-30	SAMR1	Male	101.14
SAMR1-31	SAMR1	Male	122.86
SAMR1-32	SAMR1	Male	136.86
SAMR1-33	SAMR1	Male	143.86
SAMR1-34	SAMR1	Male	115
SAMR1-35	SAMR1	Male	106.43
SAMR1-36	SAMR1	Male	105.86
SAMR1-37	SAMR1	Male	101

Supplementary Table 2. Autopsy findings in SAMP8 and SAMR1 mice.

	SAMP8	SAMR1
Probable causes of death	Unknown	Unknown
	Lymphoma	Lung tumor
	Intestinal necrosis and torsion	Ischemia
	Bronchitis and pleuritic	Lymphoma
Postmortem histopathological findings	Lymphoma and leukemia with infiltration in thymus, liver, spleen, pleura, kidney, lymph nodes and perirenal fat.	Benign cyst in peritoneum
	Steatosis in liver.	Papillary adenocarcinoma in lung
	Intestinal abscesses.	Benign tumor in cecum
	Intestinal torsion and necrosis.	Abscess in colon
	Remains of hair in the intestine. Splenomegaly.	Sarcoma
	Thymus thickened by tumor or reactive process.	Ulcer
	Brain with little mass.	Infiltration of lymphoid tumor in liver, spleen, cervical area, lymph nodes and colon
	Neurofibrillary tangles and amyloid plaques in the brain.	Neurofibrillary tangles in brain.
